# A Revised Mechanism for the Activation of Complement C3 to C3b: A MOLECULAR EXPLANATION OF A DISEASE-ASSOCIATED POLYMORPHISM*[Fn FN2]

**DOI:** 10.1074/jbc.M114.605691

**Published:** 2014-12-08

**Authors:** Elizabeth Rodriguez, Ruodan Nan, Keying Li, Jayesh Gor, Stephen J. Perkins

**Affiliations:** From the Department of Structural and Molecular Biology, Division of Biosciences, Darwin Building, University College London, Gower Street, London WC1E 6BT, United Kingdom

**Keywords:** Analytical Ultracentrifugation, Inflammation, Molecular Modeling, Neutron Scattering, Surface Plasmon Resonance (SPR), X-ray Scattering, Complement C3

## Abstract

The solution structure of complement C3b is crucial for the understanding of complement activation and regulation. C3b is generated by the removal of C3a from C3. Hydrolysis of the C3 thioester produces C3u, an analog of C3b. C3b cleavage results in C3c and C3d (thioester-containing domain; TED). To resolve functional questions in relation to C3b and C3u, analytical ultracentrifugation and x-ray and neutron scattering studies were used with C3, C3b, C3u, C3c, and C3d, using the wild-type allotype with Arg^102^. In 50 mm NaCl buffer, atomistic scattering modeling showed that both C3b and C3u adopted a compact structure, similar to the C3b crystal structure in which its TED and macroglobulin 1 (MG1) domains were connected through the Arg^102^–Glu^1032^ salt bridge. In physiological 137 mm NaCl, scattering modeling showed that C3b and C3u were both extended in structure, with the TED and MG1 domains now separated by up to 6 nm. The importance of the Arg^102^–Glu^1032^ salt bridge was determined using surface plasmon resonance to monitor the binding of wild-type C3d(E1032) and mutant C3d(A1032) to immobilized C3c. The mutant did not bind, whereas the wild-type form did. The high conformational variability of TED in C3b in physiological buffer showed that C3b is more reactive than previously thought. Because the Arg^102^-Glu^1032^ salt bridge is essential for the C3b-Factor H complex during the regulatory control of C3b, the known clinical associations of the major C3S (Arg^102^) and disease-linked C3F (Gly^102^) allotypes of C3b were experimentally explained for the first time.

## Teaser

**Background:** An understanding of the solution structure of complement C3b is essential to understand its reactivity.

**Results:** Ultracentrifugation and scattering revealed compact C3b structures in low salt and extended ones in physiological salt.

**Conclusion:** The two conformations reflect Arg^102^–Glu^1032^ salt bridge formation only in low salt.

**Significance:** The functional differences between the major C3S (Arg^102^) and C3F (Gly^102^) allotypes are explained.

## Introduction

The complement system comprises over 30 proteins arranged in a cascade as part of the innate immune response and is important for clearing immune complexes and cellular debris and for the elimination of pathogens (1, 2). C3 (complement component 3) is the most abundant complement protein, occurring at about 1.0 mg/ml (5.3 μm) in plasma and at higher levels during inflammation. All three of the complement classical, lectin, and alternative activation pathways lead to the cleavage of C3a from C3 to form active C3b, which attaches covalently to cell surfaces through a thioester bridge (1, 2). The spontaneous hydrolysis of the thioester bond in C3 leads to C3u (also known as C3_H2O_), and C3u is an activator of the alternative pathway (3) but is unable to attach to cell surfaces. C3b and C3u are regulated by the cofactor Factor H and the protease Factor I, together cleaving C3b to produce the inactive C3c and C3d fragments. Because Factor H binds to host cell surfaces and not to bacteria, host cells are protected from C3b activation (4). The two major polymorphic allotypes of C3 are C3S (with frequencies of 0.79 and 0.99 in white and Asian populations, respectively) and C3F. The nomenclature is derived from their electrophoretic mobility on agarose gels, with C3S moving slowly and C3F faster (5). C3F is associated with diseases, including IgA nephropathy, systemic vasculitis, partial lipodystrophy, membranoproliferative glomerulonephritis type II, and age-related macular degeneration. C3F may increase complement activation levels (5). In complexes with erythrocytes, C3F showed a greater affinity for mononuclear cells than C3S (6). In hemolytic activity assays, C3F showed higher alternative pathway activation than C3S due to a lowered efficiency in Factor H regulation (7).

**FIGURE 1.**
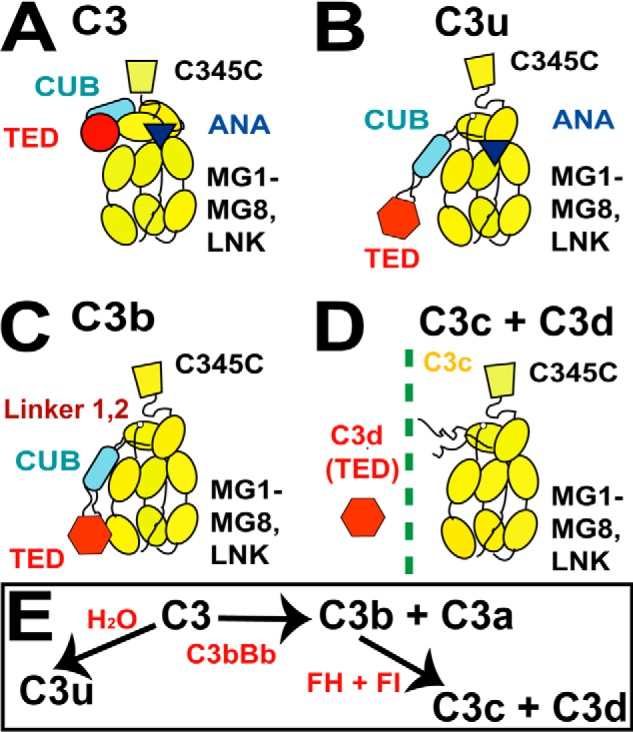
**Schematic views of the five protein structures.** *A–D*, the arrangement of the 10–13 domains of C3, C3u, C3b, and C3c depicted as schematics with the TED (*red circle* or *hexagon*), CUB (*blue rectangle*), and ANA (*dark triangle*) domains shown when present. The eight MG domains and one C345C domain are shown in *yellow. E*, summary of the relationship between the five forms of C3.

C3 belongs to the α_2_-macroglobulin protein family, members of which have a reactive thioester bond and similar domain arrangements (4). The α- and β-chains of C3 (115 and 75 kDa, respectively) are arranged as 13 domains, namely eight macroglobulin domains (MG1–MG8)^2^ and five single linker (LNK), anaphylatoxin (ANA; C3a), C1r/C1s-UEGF-BMP1 (CUB), C345c, and thioester-containing domains (TED; C3d) (8) (Fig. 1*A*). In C3, the thioester bond is buried within the TED-MG8 interface (9). During C3b activation, the release of C3a results in large conformational changes that expose the reactive TED thioester bond, which is able to bind rapidly and covalently to nucleophiles on cell surfaces (4, 10). Four crystal structures of C3b employed 50 mm NaCl as the crystal precipitant (11–14). All four structures showed that the TED domain made contact with the MG1 domain at the base of the C3b structure in a compact arrangement (Fig. 1*C*). A two-segmented structure for C3 and its homologues C4 and C5 was originally deduced using low resolution scattering modeling (15). Recent x-ray scattering and ultracentrifugation of C3u in 137 mm NaCl buffer showed that C3u has a more extended structure than in the C3b crystal structures, in which the TED and MG1 domains were well separated in C3u (Fig. 1*B*) (16). X-ray scattering of C3b in 200 mm NaCl with or without 5% glycerol suggested that C3b existed as multiple conformers with extended TED domains (17, 18). In addition, crystal structures for α_2_-macroglobulin and complement C5b in complex with C6 showed that the TED domain in these structures was also well separated from their MG1 domains (19–21).

An understanding of the different locations of the functionally crucial TED domain in the α_2_-macroglobulin protein family is essential to explain the different clinical associations of the C3S and C3F allotypes. We utilized a joint ultracentrifugation and scattering approach coupled with constrained atomistic modeling for the C3 proteins (22). Analytical ultracentrifugation established the formation of TED-mediated dimers and enabled the scattering data to be corrected for these. Improved x-ray and neutron scattering data collection in 50 and 137 mm NaCl buffers showed that the TED-MG1 domains were connected in C3b and C3u in 50 mm NaCl buffer but separated in physiological 137 mm NaCl. Crystal structures showed an Arg^102^–Glu^1032^ salt bridge between the TED and MG1 domains as part of the C3b regulatory complex with Factor H. We show that the loss of this salt bridge revises our understanding of C3b activity, making the TED domain more reactive than previously thought. Because the C3S and C3F allotypes contain Arg^102^ and Gly^102^, respectively, we can now explain for the first time the different functionality of the C3S and C3F allotypes.

## EXPERIMENTAL PROCEDURES

### 

#### 

##### Purification of C3, C3u, C3b, C3c, and C3d

C3 was purified from fresh human plasma essentially using a Q-Sepharose fast flow anion-exchange column (Amersham Biosciences) and a Mono Q 5/50 GL column (GE Healthcare) (23). The three donors were genotyped for the rs2230199 single nucleotide polymorphism (R102G) to show that all three were Arg^102^ (wild-type C3S allotype). C3u was produced by incubating C3 with 200 mm hydrazine for 2 h at 37 °C in a water bath and leaving at 4 °C overnight. C3b was produced by treating 1 mg/ml C3 in Hepes buffer (10 mm Hepes, 137 mm NaCl, 0.5 mm EDTA, pH 7.4) with 10 μg/ml trypsin (1% w/w enzyme/substrate) for 120 s at 37 °C in a water bath and then adding 40 μg/ml soybean trypsin inhibitor to stop further cleavage before transferring onto ice. Next, 20 mm iodoacetamide was added to the mixture to block the thioester, and then this was incubated in the dark at 20 °C for 30 min (13). C3b was diluted in Tris buffer (25 mm Tris, 140 mm NaCl, 0.5 mm EDTA, pH 8.0), concentrated immediately, and passed through a Superose^TM^ 6 preparation grade XK 16/60 size exclusion column. For C3c, outdated human plasma was incubated for 7 days at 37 °C in a water bath, and then C3c was purified following the same protocol as that for C3. C3u and C3b (but not C3) were active in functional assays using Factor I and Factor H (24). Recombinant C3d was expressed in *Escherichia coli* with a GST tag and purified by thrombin cleavage using a GSTrap FF 1-ml column (GE Healthcare) connected with a HiTrap Benzamidine FF (high sub) 1-ml column (GE Healthcare) (25). Western blots were performed to confirm the identity of all five proteins, using an anti-complement 3 goat polyclonal antibody (Calbiochem). The absorbance coefficients for C3, C3u, C3b, C3c, and C3d (1%, 280 nm, 1-cm path length) were calculated from their compositions to be 9.40, 9.40, 9.83, 9.21, and 13.15, respectively, assuming the presence of three high-mannose type oligosaccharides at Asn^63^, Asn^917^, and Asn^1597^ in C3 (26, 27). Molecular masses were calculated from compositions to be 189.0 kDa for C3 and C3u, 179.3 kDa for C3b, 135.7 kDa for C3c, and 34.6 kDa for C3d. All proteins were passed through a size exclusion gel filtration column (C3, C3u, C3b, and C3c in Superose 6; C3d in Superdex 200) to remove potential aggregates. For all experiments except for those in heavy water, the proteins were dialyzed into 10 mm Hepes, 50 mm NaCl, pH 7.4, or 10 mm Hepes, 137 mm NaCl, pH 7.4 (denoted as 50 mm NaCl or 137 mm NaCl, respectively, below). For heavy water dialysis, phosphate-buffered saline (PBS-^2^H_2_O) was used (137 mm NaCl, 8.1 mm Na_2_HPO_4_, 2.7 mm KCl, 1.5 mm KH_2_PO_4_, pH 7.4). Each protein was routinely checked by SDS-PAGE before and after the ultracentrifugation and scattering experiments.

##### Sedimentation Velocity Data Collection and Analyses

By analytical ultracentrifugation, sedimentation velocity data were obtained on two Beckman XL-I instruments equipped with AnTi50 rotors, using two-sector cells with column heights of 12 mm at rotor speed of 50,000 rpm. The five proteins C3, C3u, C3b, C3c, and C3d were monitored using absorbance optics at 280 nm and interference optics. For 137 mm NaCl buffer, concentration series at 20 °C were performed for C3b at concentrations between 0.25 and 1.6 mg/ml (1.4–10.3 μm) and for C3c between 0.20 and 0.98 mg/ml (1.9–7.3 μm). For 50 mm NaCl buffer, concentration series at 20 °C were performed for C3b at concentrations between 0.18 and 1.7 mg/ml (1.0–9.4 μm) and for C3c between 0.18 and 0.77 mg/ml (1.3–5.7 μm). For PBS-^2^H_2_O buffer, C3 was studied between 0.2 and 1.6 mg/ml (1.0–8.5 μm), C3u between 0.15 and 0.83 mg/ml (0.79–4.4 μm), C3b between 0.25 and 1.00 mg/ml (1.4–5.6 μm), C3c between 0.24 and 0.86 mg/ml (1.8–6.3 μm), and C3d between 0.31 and 0.92 mg/ml (9.1–26 μm).

The continuous size distribution *c*(*s*) analysis method was used to determine *s*_20,_*_w_* values using SEDFIT (version 14.1) (28, 29). The *c*(*s*) analyses provided size and shape data by directly fitting the experimental sedimentation boundaries to the Lamm equation for up to 300 interference scans for the five proteins; 25 absorbance scans for C3, C3u, C3b, and C3c; and 80 absorbance scans for C3d. The *c*(*s*) analyses were based on a fixed resolution of 200 and floated the meniscus, cell bottom, baseline, and average frictional ratio *f*/*f_o_* (where *f_o_* is the frictional coefficient of the sphere with the same volume as the hydrated glycoprotein). The starting *f*/*f_o_* values were 1.3 for C3, 1.4 for C3u, 1.38 for C3b, 1.35 for C3c, and 1.2 for C3d. Fits proceeded until the overall root mean square deviation and agreement between the observed and calculated sedimentation boundaries were satisfactory. The proportion of monomers and oligomers was quantitated by SEDFIT integration.

Buffer densities were measured at 20 °C using an Anton Paar DMA 5000 density meter for comparison with the theoretical values calculated by SEDNTERP (30). This gave densities of 1.00487 g/ml for 137 mm NaCl (theoretical, 1.00485 g/ml), 1.00195 g/ml for 50 mm NaCl (theoretical, 1.00197 g/ml), and 1.112381 g/ml for 137 mm NaCl PBS-^2^H_2_O. The viscosities of H_2_O and ^2^H_2_O were taken as 1.002 and 1.251 centipoises, respectively (31). The partial specific volumes *v* were computed as 0.739 ml/g (C3, C3u, C3b, and C3c) and 0.747 ml/g (C3d). The *v* values are affected by the protein hydration shell, being decreased in ^2^H_2_O solvent (32, 33). The reduced *v* values in ^2^H_2_O solvent were 0.728 ml/g (C3, C3u, C3b, and C3c) and 0.735 ml/g (C3d).

##### X-ray and Neutron Scattering Data Collection and Analyses

X-ray scattering data were obtained for the five proteins at 20 °C in concentration series in each of 50 and 137 mm NaCl buffers in one beam session on Beamline ID02 at the European Synchrotron Radiation Facility (Grenoble, France), operating at 6 GeV in 4-bunch mode to reduce the incident flux (34). The sample-to-detector distance was 3 meters, and the x-ray wavelength was 0.0995 nm. Potential radiation damage was eliminated by the continuous movement of the sample in a flow cell during beam exposure, the use of 10 time frames with durations of 0.3 s/frame or 0.5 s/frame in each acquisition, and visual on-line checks for radiation damage at low *Q*. No detectable effects from radiation damage were seen in the final data sets. In 137 mm NaCl, C3 was studied between 0.2 and 1.49 mg/ml (1.05–7.9 μm), C3u between 0.2 and 1.5 mg/ml (1.05–7.9 μm), C3b between 0.2 and 1.5 mg/ml (1.1–8.4 μm), C3c between 0.2 and 1.16 mg/ml (1.49–8.5 μm), and C3d between 0.2 and 1.41 mg/ml (5.7–40.2 μm). In 50 mm NaCl, C3 was studied between 0.16 and 1.25 mg/ml (0.84–6.6 μm), C3u between 0.17 and 1.29 mg/ml (0.89–6.8 μm), C3b between 0.18 and 1.40 mg/ml (1.0–7.8 μm), C3c between 0.36 and 1.46 mg/ml (2.6–10.8 μm), and C3d between 0.15 and 1.41 mg/ml (5.7–40.2 μm). Other details, including data reduction, are described elsewhere (35, 36).

Neutron scattering data were obtained on Instrument SANS2d at the ISIS pulsed neutron source at the Rutherford Appleton Laboratory (Didcot, UK) (37). Neutrons were derived from proton beam currents of ∼40 μA. Time of flight data were recorded with 4-meter collimation, a 4-meter sample-to-detector distance, a 12-mm beam diameter, and a wavelength range from 0.175 to 1.65 nm. The five proteins were measured in 2-mm-thick quartz circular banjo cells in a thermostatted rack at 20 °C. Data acquisitions lasted 1.5–3.5 h for C3 between 0.21 and 1.62 mg/ml (1.1–8.5 μm), C3u between 0.15 and 1.42 mg/ml (0.79–7.5 μm), C3b between 0.26 and 1.57 mg/ml (1.4–8.7 μm), C3c between 0.28 and 1.35 mg/ml (3.9–10.0 μm), and C3d between 0.47 and 1.13 mg/ml (13.8–33.2 μm) in PBS-^2^H_2_O. Neutron scattering data were also obtained on Instrument D22 at the Institute Laue-Langevin neutron reactor (Grenoble, France), operating at 58 MW (38). The five proteins were measured in 2-mm-thick rectangular quartz Hellma cells in a thermostatted rack at 20 °C. Data acquisitions lasted 5 min for C3 at 0.47–0.73 mg/ml (2.8–3.9 μm), C3u at 0.52–0.82 mg/ml (2.7–4.3 μm), C3b at 0.34–0.52 mg/ml (1.9–2.9 μm), C3c at 0.59–1.01 mg/ml (4.3–7.4 μm), and C3d at 0.33 mg/ml (9.7 μm) in PBS-^2^H_2_O.

In a given solute-solvent contrast, the radius of gyration (*R_g_*) corresponds to the mean square distance of scattering elements from their center of gravity and is a measure of structural elongation. Guinier plots at low *Q* give the *R_g_* and scattering at zero angle *I*(0) from the following expression (39).

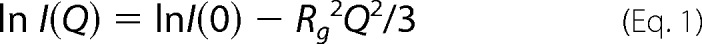
 This expression is valid in a *Q*·*R_g_* range up to 1.5. The *I*(0)/*c* value (*c* is the protein concentration in mg/ml) is proportional to the relative molecular mass *M*_r_. If the structure is elongated, the radius of gyration of the cross-sectional structure (*R_xs_*) and the mean cross-sectional intensity at zero angle (*I*(*Q*)·*Q*) are obtained from *Q* values larger than those used for *R_g_* analysis.

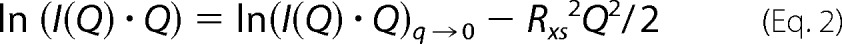
 The *R_xs_* value monitors the elongation of its cross-section shape along its longest axis. The Guinier analyses were performed using an interactive PERL script program SCTPL7 (J. T. Eaton and S. J. Perkins) on Silicon Graphics OCTANE workstations.

Indirect transformation of the *I*(*Q*) curve measured in reciprocal space into real space gives the distance distribution function *P*(*r*) and was carried out using the program GNOM (40).

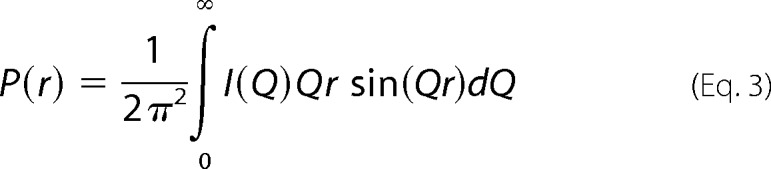

*P*(*r*) corresponds to the distribution of distances *r* between volume elements. This offers an alternative calculation of the *R_g_* and *I(0*) values that is based on the full scattering curve *I(Q*) and not that at low *Q*. It also gives the most frequently occurring distance *M* and the maximum dimension of the macromolecule *L*. For the five proteins, the x-ray curves utilized up to 340 data points for *Q* values between 0.16 and 1.50 nm^−1^. The neutron SANS2d curves utilized up to 50 data points for *Q* values between 0.20 and 2.1 nm^−1^, whereas the neutron D22 curves utilized up to 109 data points for *Q* values between 0.09 and 1.96 nm^−1^. Other details are described elsewhere (24, 35, 36).

##### Scattering and Sedimentation Modeling of C3, C3u, C3b, C3c, and C3d

The scattering modeling of human C3, C3c, and C3d utilized their crystal structures (Protein Data Bank codes 2A73, 2A74, and 1C3D, respectively). To calculate their scattering curves, the C3, C3c, and C3d crystal structures were converted into Debye spheres. A cube side length of 0.54 nm for all three structures with a cut-off of four atoms gave totals of 2047, 1408, and 367 spheres for the neutron modeling that were within 1% of their unhydrated volumes of 1557, 1112, and 224 nm^3^, respectively. The hydration shell detectable by x-ray scattering was incorporated by adding extra spheres to the surface of the unhydrated sphere model using HYPRO (41), based on a hydrated volume of 0.3 g of H_2_O/g of glycoprotein. The optimal totals of hydrated spheres were 2047, 1408, and 367, respectively, for C3, C3c, and C3d. The scattering curve *I(Q*) was calculated using the Debye equation adapted to spheres (42). Details are given elsewhere (33, 43). The four C3b crystal structures used here have Protein Data Bank codes of 2I07, 2WIN, 2WII, and 2ICF.

The constrained scattering modeling of C3, C3u, and C3b followed previous procedures (16). The x-ray modeling for C3 and C3u was extended to the neutron modeling. C3u and C3b were each considered in terms of a C3c region connected to the CUB-TED domain pair by a conformationally variable linker between the MG8 and CUB domains (Fig. 1*C*). The same 4650 trial models previously generated from the C3b crystal structure (Protein Data Bank code 2I07) showed linker lengths between 0.59 and 2.72 nm. The unhydrated models for C3b and C3u contained 1478 spheres, and the hydrated models contained 1945 spheres. The C3 modeling was considered in terms of varying the two linkers between the CUB-MG7 and CUB-TED domain pairs, to result in 8000 trial C3 models. The C3 models optimally contained 1559 spheres (unhydrated) and 2050 spheres (hydrated). Steric overlap between the C3c region and the CUB and/or TED domains was assessed using the number of spheres *n* following grid transformation. Models with less than 95% of *n* were discarded. The *R_g_* and *R_xs_* values of the remaining models were required to be within ±5% of their experimental values. Models that passed the *n*, *R_g_*, and *R_xs_* filters were ranked using a *R*-factor goodness of fit parameter defined by analogy with protein crystallography. The curve fits used experimental curves with *Q* ranges extending up to 1.50, 2.1, and 1.96 nm^−1^ for C3b, C3, and C3u, respectively. The 10 best fit C3b models from 50 and 137 mm NaCl buffers and that for C3u in 50 mm NaCl were deposited in the Protein Data Bank with the accession codes 4MRJ, 4MRK, and 4MRL, respectively. That for C3u in 137 mm NaCl had been deposited with the Protein Data Bank code 3MMQ.

Sedimentation coefficients *s*_20,_*_w_* for the C3, C3b, C3c, and C3d crystal structures and for the C3, C3u, and C3b best fit scattering models were calculated directly from the atomic coordinates using the HYDROPRO shell-modeling program (44). The default value of 0.31 nm for the atomic element radius for all atoms was used to represent the hydration shell.

##### Surface Plasmon Resonance with Mutant C3d(A1032)

Site-directed mutagenesis was performed using the QuikChange^TM^ site-directed mutagenesis kit (Agilent Technologies). The C3d coding region was amplified by PCR using forward 5′-CTGGATGAAACGGCGCAGTGGGAGAAG-3′ and reverse 5′- CTTCTCCCACTGCGCCGTTTCATCCAG-3′ primers that incorporated the E1032A change. The PCR products were treated with DpnI restriction enzyme to remove methylated parental DNA, and the plasmid was transformed into XL-1 blue-competent cells (Agilent Technologies). Following confirmation of the correct sequence by DNA sequencing (Eurofins, MWG Operon, London, UK), the mutant recombinant DNA was transformed using heat shock into *E. coli* BL21 cells for protein expression (Novagen, Merck). Surface plasmon resonance binding studies were performed on a Biacore X100 system (GE Healthcare). C3c was immobilized to the flow cell of a carboxylated dextran (CM5) research grade sensor chip via a standard amine coupling procedure according to the manufacturer's protocol. 10 μg/ml C3c in 10 mm acetate buffer (pH 4.5) was injected over flow cell 2 until the appropriate level of response units for kinetic analyses was attained. A control was prepared identically on flow cell 1 but without immobilizing C3c. Binding and steady state analyses with wild type and mutant C3d were performed at 25 °C using appropriate Biacore X100 wizards at a flow rate of 30 μl/min. Regeneration after each run was achieved by pulsing 10 mm acetate buffer, 2 m NaCl (pH 7.1) across both flow cells twice for 30 s. The analyte was passed over the chip surface in 50 mm NaCl or 137 mm NaCl HEPES buffers. For the steady state analysis of wild type C3d binding to C3c, 0.23–1.55 mg/ml (7–45 μm) C3d was passed over the sensor surface in 50 mm NaCl and 0.38–2.27 mg/ml (11–66 μm) in 137 mm NaCl, respectively. For the mutant C3d, 0.24–0.8 mg/ml (7–23.1 μm) in 50 and 137 mm NaCl buffers was injected over immobilized C3c.

## RESULTS

### 

#### 

##### Sedimentation Velocity Analyses

**FIGURE 2.**
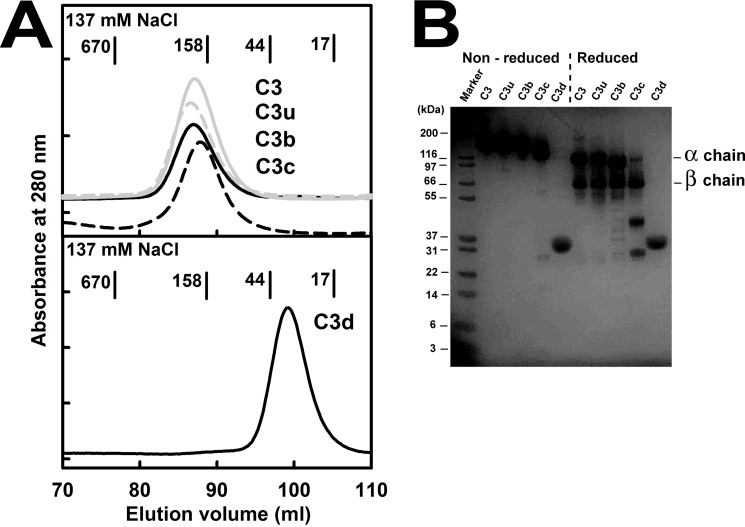
**Purification of the five proteins.** *A*, the elution profiles in 137 mm NaCl buffer of C3 (*continuous gray line*), C3u (*dashed gray line*), C3b (*continuous black line*), and C3c (*dashed black line*) are shown at the *top*, and that for C3d is shown at the *bottom. B*, non-reduced and reduced SDS-PAGE analyses of the five proteins in 137 mm NaCl buffer. The α and β chains of C3, C3u, and C3b are *labeled*, together with the masses of the protein markers.

C3, C3u, C3b, C3c, and C3d were studied in concentration ranges between 1 and 10 μm, these being comparable with the physiological plasma C3 concentration of 1.0 mg/ml (5.3 μm) (2). During gel filtration, the five purified proteins C3, C3u, C3b, C3c, and C3d (see “Experimental Procedures”) each eluted as a single symmetric peak in 137 mm buffer (Fig. 2*A*). Before and after experiments, C3, C3u, and C3b each migrated as a single band in non-reducing SDS-PAGE and as two bands that correspond to their α and β chains in reducing SDS-PAGE (Fig. 2*B*). C3c migrated as a single band in non-reducing SDS-PAGE and three bands that correspond to the cleaved α chain and the β chain in reducing SDS-PAGE. C3d migrated as one band in both reducing and non-reducing SDS-PAGE.

**FIGURE 3.**
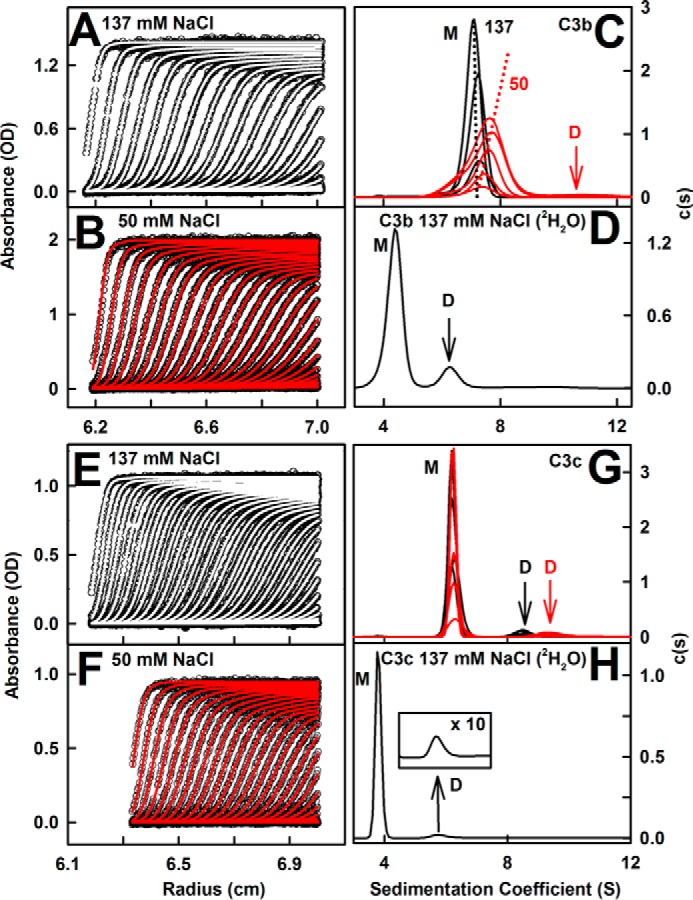
**Sedimentation distribution analyses for C3b and C3c.** *A* and *B*, absorbance boundary fits for C3b in 137 and 50 mm NaCl buffers. For clarity, only every eighth absorbance boundary is shown from 200 scans. Interference data are not shown. *C*, the corresponding *c*(*s*) distributions for 0.2–1.57 mg/ml C3b in 137 mm NaCl (*black*) and 0.18–1.7 mg/ml C3b in 50 mm NaCl (*red*). The *two dashed lines* highlight the dependence of *s* on increasing C3b concentration (*M*, monomer; *D*, dimer). *D*, the *c*(*s*) distribution for 1.0 mg/ml C3b in 137 mm NaCl in PBS-^2^H_2_O. *E* and *F*, the corresponding boundary fits for C3c in 137 and 50 mm NaCl are shown. *G*, the *c*(*s*) distributions for 0.2–0.97 mg/ml C3c in 137 mm NaCl (*black*) and 0.18–0.80 mg/ml C3c in 50 mm NaCl (*red*). *H*, the *c*(*s*) distribution for 0.86 mg/ml C3c in 137 mm NaCl in PBS-^2^H_2_O.

**FIGURE 4.**
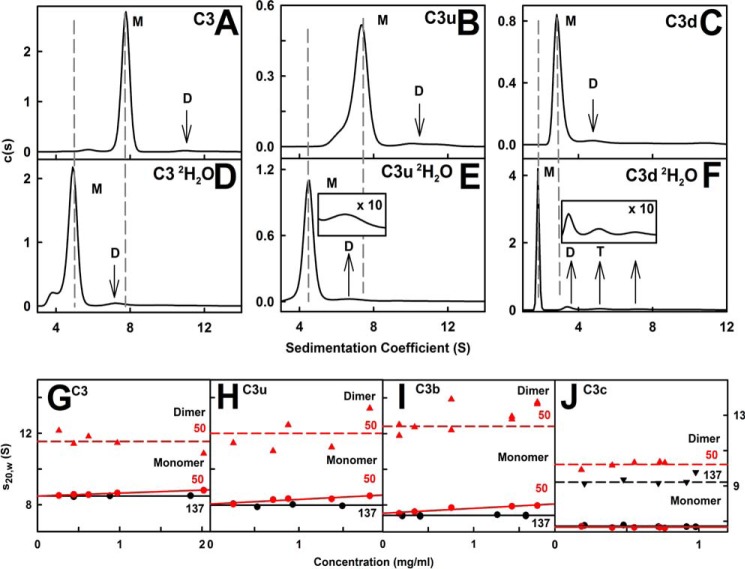
**Summary of the sedimentation analyses for the five proteins.** *A–C*, the *c*(*s*) distributions for 1.72 mg/ml C3, 0.39 mg/ml C3u, and 0.55 mg/ml C3d in 137 mm NaCl buffer in H_2_O. *D–F*, the *c*(*s*) distributions for 1.62 mg/ml C3, 0.83 mg/ml C3u, and 0.91 mg/ml C3d in 137 mm NaCl in ^2^H_2_O. The monomer (*M*), dimer (*D*), and trimer/tetramer (*T*) peaks are *labeled*. The *dashed lines* indicate the change in the monomer *s* values on going from light to heavy water. *G–J*, the *s*_20,_*_w_* values for the four monomers of C3, C3u, C3b, and C3c in 50 mm NaCl (*red*) were fitted by linear regression to give the *s*_20,*w*_^0^ values. Those in 137 mm NaCl are shown as their mean. Those for dimers are shown by *dashed lines*. The *s*_20,*w*_^0^ values are summarized in Table 1.

Analytical ultracentrifugation is used to observe macromolecular sedimentation under high centrifugal force in order to determine their masses and shapes (45). The main advantage of ultracentrifugation is the detection of different species in a sample from the peaks in size distribution analyses *c*(*s*). Previous *c*(*s*) data for C3, C3u, and C3d in 50 and 137 mm NaCl buffers (16, 46) were extended to include C3b and C3c in the same buffers. Good absorbance and interference boundary fits were obtained (Fig. 3, *A*, *B*, *E*, *F*), and new data for C3, C3u, and C3d confirmed previous results (Fig. 4, *A–C*). (i) In 137 mm NaCl buffer, C3b showed primarily a monomer peak with a mean *s*_20,_*_w_* value of 7.40 S and a molecular mass of 179 kDa (Figs. 3*C* (*M*) and Fig. 4*I*). A small C3b dimer peak (2% of the total) was seen at 11.4 S with the expected molecular mass of 331 kDa. C3c showed a monomer peak at 6.53 S and 135 kDa and a small C3c dimer peak (6%) at 9.2 S and 250 kDa (Figs. 3*G* (*M* and *D*) and 4*J*). No concentration dependences were observed. Peak integrations gave estimated *K_D_* values for the monomer-dimer equilibrium of 55 ± 20 μm for C3b and 60 ± 15 μm for C3c. (ii) In 50 mm NaCl buffer, the C3b monomer peak showed a concentration dependence (Fig. 3*C*), attributable to fast exchange with C3b dimers. C3 and C3u also showed this fast exchange (16). Upon extrapolation of the *s*_20,_*_w_* values to zero concentration, the *s*_20,*w*_^0^ value for monomeric C3b was 7.60 S (Fig. 4*I*) with a mass of 183 kDa. A small C3b dimer (4%) was visible at 12.4 S with a mass of 326 kDa (Fig. 3*C*). The 0.19 S increase in 50 mm NaCl buffer suggested that C3b is more compact in low salt. The C3c monomer and dimer peaks were unchanged at 6.53 and 10.3 S (Fig. 3*G*). The estimated monomer-dimer *K_D_* values were comparable at 40 ± 20 μm for C3b and 50 ± 15 μm for C3c.

C3, C3u, and C3d showed well defined monomer peaks in 137 mm NaCl buffer (Fig. 4, *A–C*). Our improved data analyses gave *s*_20,_*_w_* values of 8.49, 8.03, and 3.03 S, respectively, in agreement with previous results (16, 46). Dimers were seen at 12.1 S (3%), 11.9 S (8%), and 5.9 S (10%), respectively. For 137 mm NaCl, the estimated monomer-dimer *K_D_* values were 70 ± 10 μm for C3 and 45 ± 15 μm for C3u. In 50 mm NaCl, C3 and C3u showed concentration dependences as before (16). The extrapolated *s*_20,*w*_^0^ values were 8.50 S (C3) and 8.10 S (C3u) (Fig. 4) with masses of 190 and 175 kDa, respectively. For 50 mm NaCl, the estimated monomer-dimer *K_D_* values were 45 ± 10 μm for C3, 40 ± 10 μm for C3u, and 25 ± 10 μm for C3d. The similar salt dependences in 50 mm NaCl for both C3b and C3u, but not for C3c, implicated the TED domain (equivalent to C3d) as being responsible for the fast dimerization of C3b and C3u. The oligomerization of C3d in 50 mm NaCl but not in 137 mm NaCl supports this conclusion (Fig. 2 in Ref. 46).

Ultracentrifugation in ^2^H_2_O confirmed dimerization because this solvent promotes protein self-association. The monomers showed *s* values of 4.78 S (C3), 4.53 S (C3u), 4.53 S (C3b), 3.79 S (C3c), and 1.78 S (C3d) (Figs. 3 (*D* and *H*) and 4 (*D* and *E*)). Correction for the buffer density and viscosity of ^2^H_2_O and reduced protein *v* values (see “Experimental Procedures”) 8.50 S (C3), 8.08 S (C3u), 7.53 S (C3b), 6.54 S (C3c), and 3.23 S (C3d) were in good agreement with the light water values. Increased amounts of dimers were observed in 137 mm NaCl in ^2^H_2_O buffer. Their *s* values were 7.41 S (C3), 7.36 S (C3u), 6.60 S (C3b), 5.76 S (C3c), and 3.53 S (C3d) plus additional C3d peaks at 5.26 S and 7.76 S (Figs. 3 (*D* and *H*) and 4 (*D–F*)). Their conversion to *s*_20,_*_w_* gave 12.47 S (C3), 12.04 (C3u), 10.61 S (C3b), 9.64 S (C3c), and 6.23 S (C3d). The dimer proportions increased to 5% (C3), 15% (C3u), 14% (C3b), 7% (C3c), and 12% (C3d). The doubling of dimer formation to 14–15% for C3u and C3b, in contrast to the almost unchanged dimer of 2–4% for C3 and 6–7% for C3c, indicated the assignment of the fast dimerization site in C3u and C3b to the exposed TED (C3d) domain.

##### X-ray and Neutron Scattering Analyses

**FIGURE 5.**
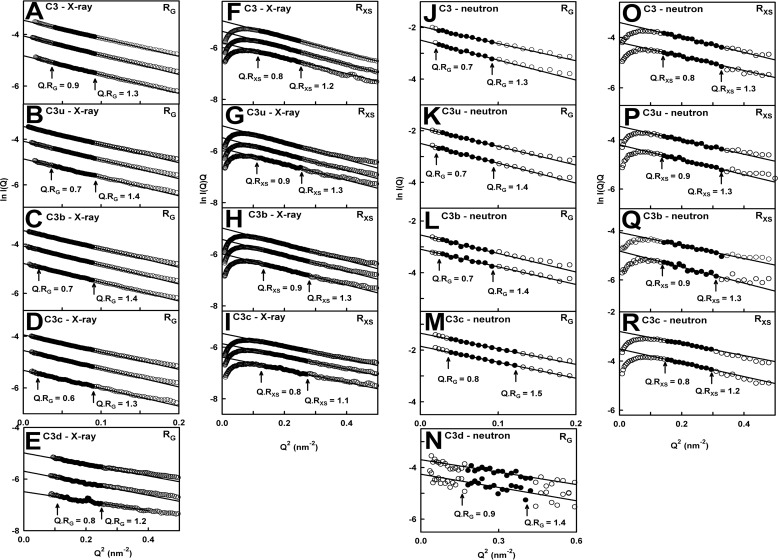
**Guinier *R_g_* and *R_xs_* analyses for the five proteins.** *A–I*, in the x-ray analyses, the C3, C3u, and C3b concentrations in 137 mm NaCl buffer ranged between 0.5 and 1.5 mg/ml (from *bottom* to *top*, as shown); those for C3c ranged between 0.4 and 1.1 mg/ml; and those for C3d ranged between 0.5 and 1.4 mg/ml. The *Q* ranges used for the *R_g_* fits were 0.18–0.30 nm^−1^ for C3; 0.14–0.30 nm^−1^ for C3u, C3b, and C3c; and 0.35–0.55 nm^−1^ for C3d. Those for the *R_xs_* fits were 0.35–0.50 nm^−1^ for all four proteins. The *filled circles* represent the data used to determine the *R_g_* and *R_xs_* values. Their values were measured within satisfactory *Q*·*R_g_* and *Q*·*R_xs_* ranges, as shown. *J–R*, in the neutron analyses, the C3 concentrations were 0.75 and 0.47 mg/ml; those for C3u were 0.82 and 0.52 mg/ml; those for C3b were 0.52 and 0.34 mg/ml; those for C3c were 0.91 and 0.53 mg/ml; and those for C3d were 0.55 and 0.34 mg/ml. The D22 data sets correspond to ^2^H_2_O buffer. The *Q* ranges used for the *R_g_* fits were 0.16–0.30 nm^−1^ for C3, C3u, and C3b; 0.2–0.35 nm^−1^ for C3c; and 0.4–0.6 nm^−1^ for C3d. Those for the *R_xs_* fits were 0.35–0.50 nm^−1^ for all four proteins. The *filled circles* represent the data used to determine the *R_g_* and *R_xs_* values.

Small angle x-ray and neutron scattering is a diffraction technique that determines overall macromolecular structures in solution (19). X-rays were used to examine the hydrated proteins in 50 and 137 mm NaCl buffers. Neutrons were used to examine their unhydrated protein structures in 137 mm NaCl ^2^H_2_O buffer (33). The x-ray and neutron Guinier analyses showed high quality linear plots in two distinct *Q* ranges. The lowest *Q* values gave the *R_g_* values, which monitor the degree of elongation (Fig. 5, *A–E* and *J–N*). At larger *Q* values, the *R_xs_* values measure the mean cross-sectional dimensions (Fig. 5, *F–I* and *O–R*). The Guinier *I*(*0*)/*c* values are proportional to the relative molecular masses (39, 47).

**FIGURE 6.**
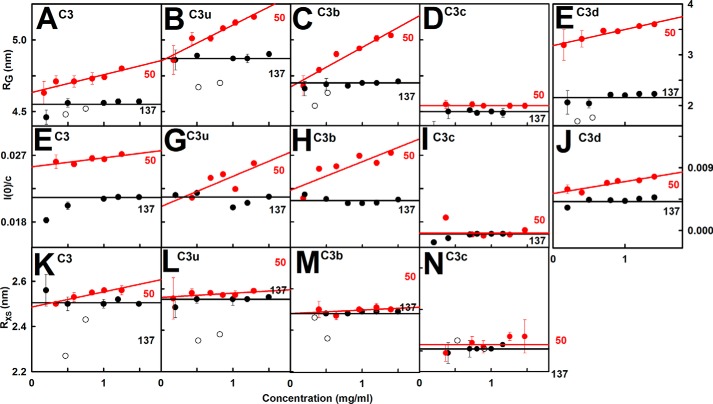
**Concentration dependence of the Guinier values.** Each x-ray value is the mean of four measurements. The neutron values from D22 correspond to single measurements. *A–E*, x-ray *R_g_* values are shown for 137 mm (*black*) and 50 mm NaCl (*red*) buffers. The *pairs* of *open symbols* correspond to the neutron *R_g_* values. For 137 mm NaCl, the *line* denotes the mean value. For 50 mm NaCl, linear regression gave the mean ± S.D. *F–J*, corresponding x-ray *I*(*0*)/*c* analyses. *K–N*, corresponding x-ray and neutron *R_xs_* values.

The *R_g_* values depended on the buffer in use as follows. (i) In 137 mm NaCl buffer, the x-ray *R_g_* values for C3, C3u, C3b, C3c, and C3d were unchanged with concentration at 4.54, 4.87, 4.71, 4.47, and 2.15 nm, respectively (Fig. 6, *A–E*, and Table 1). The largest *R_g_* values for C3u and C3b reflected their more elongated structures. The *I*(*0*)/*c* values of 0.0213, 0.0213, 0.0207, 0.0163, and 0.0044 for C3, C3u, C3b, C3c, and C3d (Fig. 6, *F–J*) were in proportion to their molecular masses (189, 189, 179, 135, and 34 kDa, respectively). The *R_xs_* values of C3, C3u, and C3b were similar at 2.50, 2.54, and 2.49 nm, respectively, and that for C3c was lower at 2.21 nm, as expected (Fig. 6, *K–N*). (ii) In 50 mm NaCl buffer, the Guinier parameters for C3, C3u, C3b, and C3d (but not C3c) showed concentration dependences (Fig. 6). Extrapolation of the *R_g_* values to zero concentration gave 4.63, 4.85, 4.67, 4.54, and 3.18 nm, respectively, all four being close to the 137 mm NaCl values except for C3d (Table 1). The extrapolated *I*(*0*)/*c* values of 0.025, 0.021, 0.022, 0.0165, and 0.005, respectively, were similar to those in 137 mm NaCl buffer (Fig. 6, *F–J*). The extrapolated *R_xs_* values for C3, C3u, and C3b were also the same as those in 137 mm NaCl (Table 1). (iii) In 137 mm NaCl buffer in heavy water, the five proteins aggregated above 1 mg/ml. Upon halving the concentrations with fresh samples, the Guinier analyses gave high quality linear fits for the *R_g_* and *R_xs_* analyses (Fig. 5, *J–N*). The *R_g_* and *R_xs_* values were slightly below the x-ray values by reason of the nearly invisible hydration shell (33). The *R_g_* values were 4.32 nm (C3), 4.69 nm (C3u), 4.53 nm (C3b), 4.22 nm (C3c), and 1.73 nm (C3d) (Table 1). The *R_xs_* values were 2.35 nm (C3), 2.37 nm (C3u), 2.38 nm (C3b), and 2.23 nm (C3c).

**TABLE 1:** **Experimental scattering and sedimentation data for C3, C3u, C3b, C3c, and C3d**

Protein (NaCl concentration)	*R_g_* (x-ray)*^a^*	*R_XS_* (x-ray)	*L* (x-ray)	*R_G_* (neutron)*^a^*	*R_XS_* (neutron)	*L* (neutron)	*s*_20, *w*_^0^
	*nm*	*nm*	*nm*	*nm*	*nm*	*nm*	*S*
C3 (137 mm)	4.54 ± 0.04	2.50 ± 0.03	16	4.32 ± 0.01	2.35 ± 0.09	15	8.49 ± 0.03*^b^*
	4.73 ± 0.01			4.62 ± 0.01			
C3u (137 mm)	4.87 ± 0.02	2.54 ± 0.02	16	4.69 ± 0.02	2.37 ± 0.02	16	8.03 ± 0.09*^b^*
	4.97 ± 0.06			4.78 ± 0.12			
C3b (137 mm)	4.71 ± 0.016	2.49 ± 0.04	16	4.53 ± 0.10	2.38 ± 0.02	16	7.40 ± 0.04
	4.81 ± 0.02			4.77 ± 0.03			
C3c (137 mm)	4.47 ± 0.05	2.21 ± 0.02	14	4.22 ± 0.02	2.23 ± 0.02	14	6.53 ± 0.06
	4.56 ± 0.02			4.31 ± 0.04			
C3d (137 mm)	2.15 ± 0.09	n.a.*^c^*	6	1.73 ± 0.04	n.a.	7	3.03 ± 0.02
	2.21 ± 0.03			2.15 ± 0.14			
C3 (50 mm)	4.63 ± 0.12	2.49 ± 0.12	15–19	n.a.	n.a.	n.a.	8.50 ± 0.16*^b^*
	4.87 ± 0.21						
C3u (50 mm)	4.85 ± 0.22	2.55 ± 0.22	17–20	n.a.	n.a.	n.a.	8.10 ± 0.19*^b^*
	5.34 ± 0.23						
C3b (50 mm)	4.67 ± 0.22	2.49 ± 0.17	17–20	n.a.	n.a.	n.a.	7.60 ± 0.05
	5.18 ± 0.24						
C3c (50 mm)	4.54 ± 0.05	2.23 ± 0.03	14	n.a.	n.a.	n.a.	6.53 ± 0.06
	4.59 ± 0.05						
C3d (50 mm)	3.18 ± 0.31	n.a.	6–12	n.a.	n.a.	n.a.	3.0 ± 0.3*^d^*
	2.99 ± 0.54						

*^a^* The first *R_g_* value is from the Guinier analyses; the second *R_g_* value is from the *P*(*r*) analyses.

*^b^* Our previous study (16) had reported *s* values of 7.85 ± 0.05 S for C3 and 7.44 ± 0.07 S for C3u in 137 mm NaCl. These should have been reported as *s*_20, *w*_^0^ values of 8.49 ± 0.03 and 8.03 ± 0.09 S, respectively. In addition, the previous *s* values of 8.02 ± 0.12 S for C3 and 7.66 ± 0.19 S for C3u in 50 mm NaCl should have been reported as *s*_20, *w*_^0^ values of 8.50 ± 0.16 and 8.10 ± 0.19 S, respectively.

*^c^* n.a., not available.

*^d^* From Ref. 46.

**FIGURE 7.**
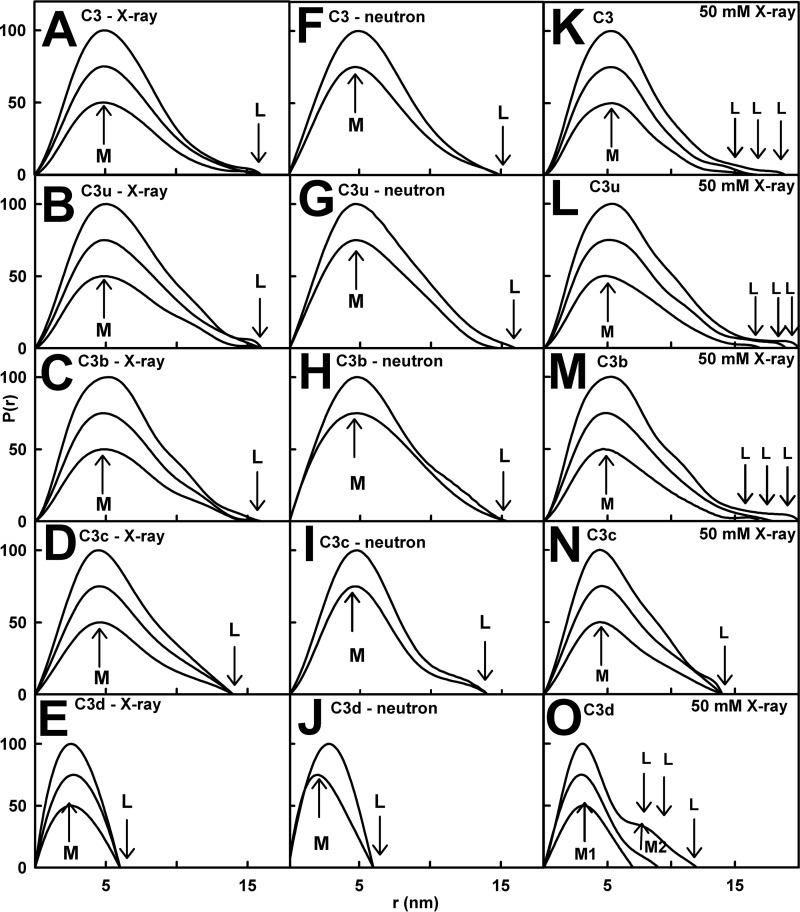
**Distance distribution functions *P*(*r*) for the five proteins.** *A–E*, for C3, C3u, and C3b in 137 mm NaCl by x-rays, the maximum *M* is at 5.0 nm; *M* for C3c is at 4.5 nm; and *M* for C3d is at 2.5 nm. For C3, C3u, and C3b, the maximum length *L* is at 16 nm; *L* for C3c is at 14 nm; and *L* for C3d is at 7 nm. *F–J*, the neutron data in 137 mm NaCl showed similar *M* and *L* values for the five proteins. *K–O*, the x-ray data in 50 mm NaCl showed *L* values for C3 of 15 nm (0.5 mg/ml), 16 nm (0.8 mg/ml), and 19 nm (1.25 mg/ml). For C3u, the *L* values increased from 16 to 20 nm between 0.17 and 1.29 mg/ml. For C3b, the *L* values increased from 17 to 20 nm between 0.4 and 1.40 mg/ml. For C3c, the *L* values of 14 nm were unchanged between 0.16 and 1.25 mg/ml. C3d showed *L* values of 6 nm (0.15 mg/ml), 9 nm (0.5 mg/ml), and 12 nm (1.41 mg/ml) and two peaks denoted *M1* and *M2* at 1.41 mg/ml.

The distance distribution function *P(r*) leads to the overall length (*L*) at large *r* and the most frequent interatomic distance *M*. The *R_g_* and *I*(0)/*c* values from *P*(*r*) agreed with those from the Guinier analyses (Table 1). The *L* values for C3, C3u, C3b, C3c, and C3d in 137 mm NaCl were 16, 16, 16, 14, and 6 nm, respectively (Fig. 7). In 50 mm NaCl, the *L* values for C3, C3u, and C3b increased from 16 nm at 0.5 mg/ml to 20 nm at 1.5 mg/ml and from 6 to 12 nm for C3d, reflecting dimerization (Fig. 7). C3c showed no change in *L*. The neutron *P*(*r*) analyses gave *L* values similar to the x-ray values or slightly less (Fig. 7). The *M* values of C3, C3u, and C3b were all 5 nm, C3c was 4.5 nm, and C3d was 2.5 nm, these being unchanged with concentration or buffer by x-rays or neutrons.

##### Comparison with known C3, C3u, C3b, C3c, and C3d Structures

**FIGURE 8.**
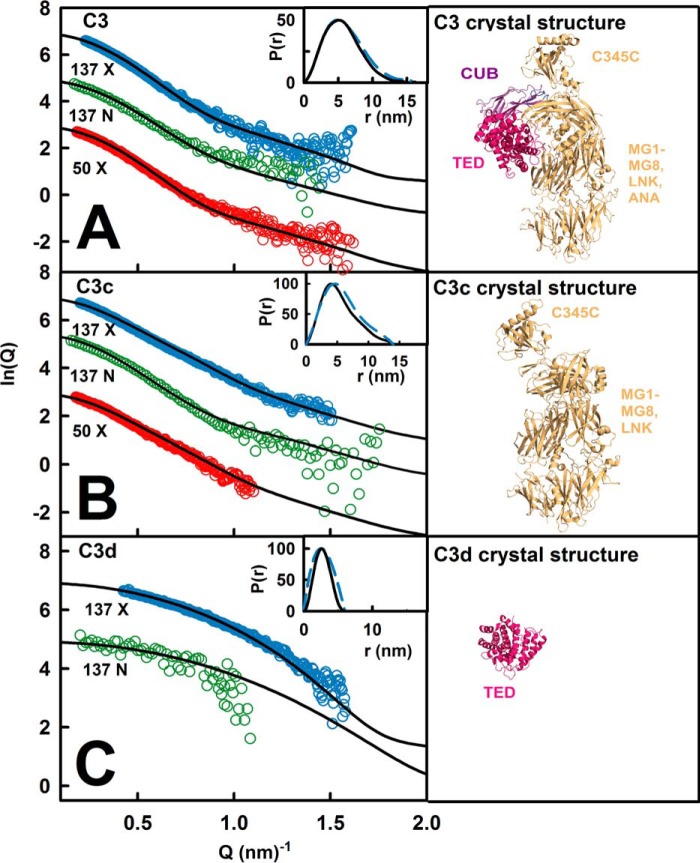
**Scattering curve fits using the C3, C3c, and C3d crystal structures.** *A*, the three experimental curves for C3 (concentrations of 0.73–1.49 mg/ml) are shown in *circles*, and the *black line* is based on the C3 crystal structure. *B*, the experimental curves for C3c (1.01–1.16 mg/ml) are shown in *circles*, and the *black line* is based on the C3c crystal structure. *C*, the experimental curves for C3d/TED (0.50–0.59 mg/ml) are shown in *circles*, and the *black line* is based on the C3d crystal structure.

The five molecular structures were compared with their three scattering curves in different buffers (Fig. 1 and Table 2*A*). For 137 mm NaCl, the C3, C3c, and C3d crystal structures gave good fits (Fig. 8). The *R*-factors (goodness of fit) were low, at 3.2–3.9% for x-rays and 3.9–5.1% for neutrons (Table 2*A*). The four C3b crystal structures gave *R*-factors of 3.3 ± 0.1% (x-rays) and 6.6 ± 0.4% (neutrons). The C3u solution structure gave *R*-factors of 3.3% (x-rays) and 4.5% (neutrons). For 50 mm NaCl, because ultracentrifugation showed that dimers were present, it was necessary to extrapolate the full curves to zero concentration to eliminate the effect of dimers. The resulting C3 and C3c *R*-factors were 3.8–3.9%, similar to those in 137 mm NaCl, and C3u gave a similar *R*-factor of 3.3%. Of importance was the much reduced *R*-factor of 1.5% for C3b, showing that the curve fit was improved. Other comparisons using *R_g_* values calculated from molecular structures were less precise, being sensitive to small amounts of dimer (Fig. 6 and Table 1). For all five proteins except one, the experimental *R_g_* was between 0.04 and 0.59 nm larger than the *R_g_* values from molecular structures (Table 2*A*), suggesting that trace amounts of dimers perturbed the Guinier fits. The exception was C3d in 50 mm NaCl, where the experimental *R_g_* of 2.99–3.18 nm showed a large difference from the crystal structure value of 2.11 nm, indicating that dimers were still present.

**TABLE 2:**
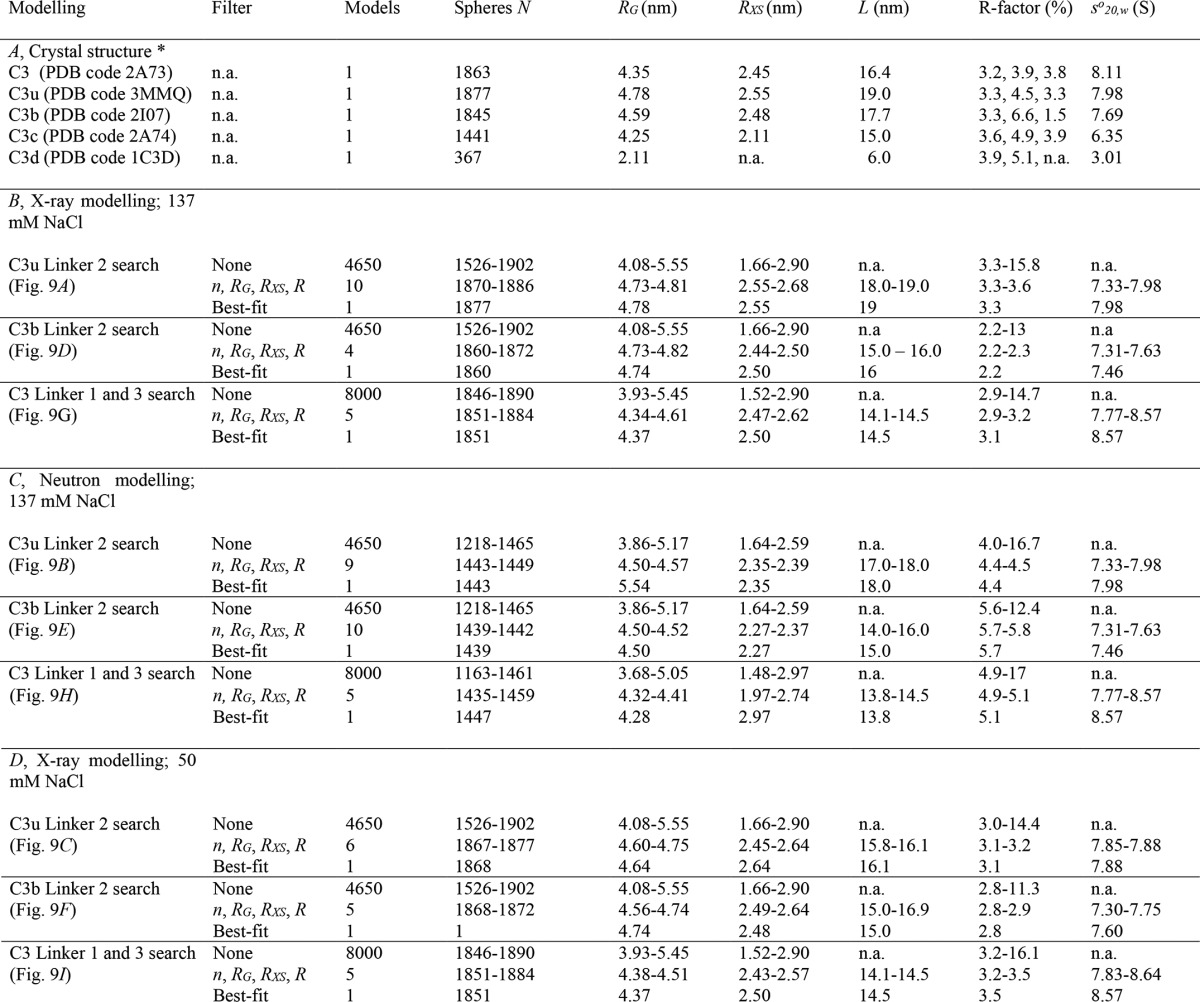
**Scattering and sedimentation parameters for C3, C3u, C3b, C3c, and C3d calculated from molecular modeling fits**

* In *A*, the three R-factors are reported in the order of *B* (x-ray modeling; 137 mm NaCl), *C* (neutron modeling; 137 mm NaCl), and *D* (x-ray modeling; 50 mm NaCl).

n.a., not applicable or not available.

The *s*_20,*w*_^0^ calculations from molecular structures suggested that C3u and C3b were more compact in 50 mm NaCl and more extended in 137 mm NaCl, although the precision of the calculation was only ± 0.21 S (48). C3u with its extended TED-MG1 domain pair arrangement gave a solution structural value of 7.98 S (Table 2*A*), in better agreement with the experimental *s*_20,*w*_^0^ of 8.03 S in 137 mm NaCl than that of 8.10 S in 50 mm NaCl (Table 1). C3b with its compact TED-MG1 domain pair arrangement gave a crystal structure value of 7.69 S, in better agreement with the *s*_20,*w*_^0^ of 7.60 S in 50 mm NaCl than that of 7.40 S in 137 mm NaCl. For C3, the crystal structure value of 8.11 S was comparable with the experimental *s*_20,*w*_^0^ values of 8.49–8.50 S in both 50 mm and 137 mm NaCl. For C3c, the crystal structure value of 6.35 S agreed with the experimental *s*_20,*w*_^0^ of 6.53 S in both 50 and 137 mm NaCl. For C3d, the crystal structure value of 3.01 S agreed with the experimental *s*_20,*w*_^0^ of 3.0 S in 50 and 137 mm NaCl (46).

##### Atomistic Modeling Searches for C3u, C3b, and C3

**FIGURE 9.**
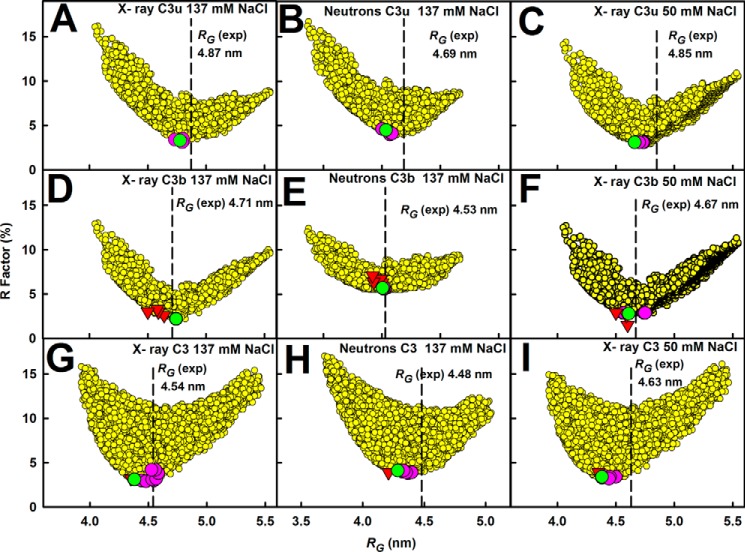
**Constrained modeling of C3u, C3b, and C3 in three buffers.** The *R*-factors for the 4650 conformationally randomized models for C3u (*A–C*) and C3b (*D–F*) and 8000 randomized models for C3 (*G–I*) are compared with their corresponding *R_g_* values. The three buffers were 137 mm NaCl in light water for x-rays (*A*, *D*, and *G*); 137 mm NaCl in heavy water for neutrons (*B*, *E*, and *H*); and 50 mm NaCl in light water for X-rays (*C*, *F*, and *I*). In each *panel*, the *vertical dashed line* corresponds to the experimental *R_g_* value. The *R*-factors for the 10 best fit models are shown in *pink*, and the best fit of these is shown in *green*. The *R*-factors for the C3b and C3 crystal structures are denoted by *inverted red* triangles in *D–I*.

**FIGURE 10.**
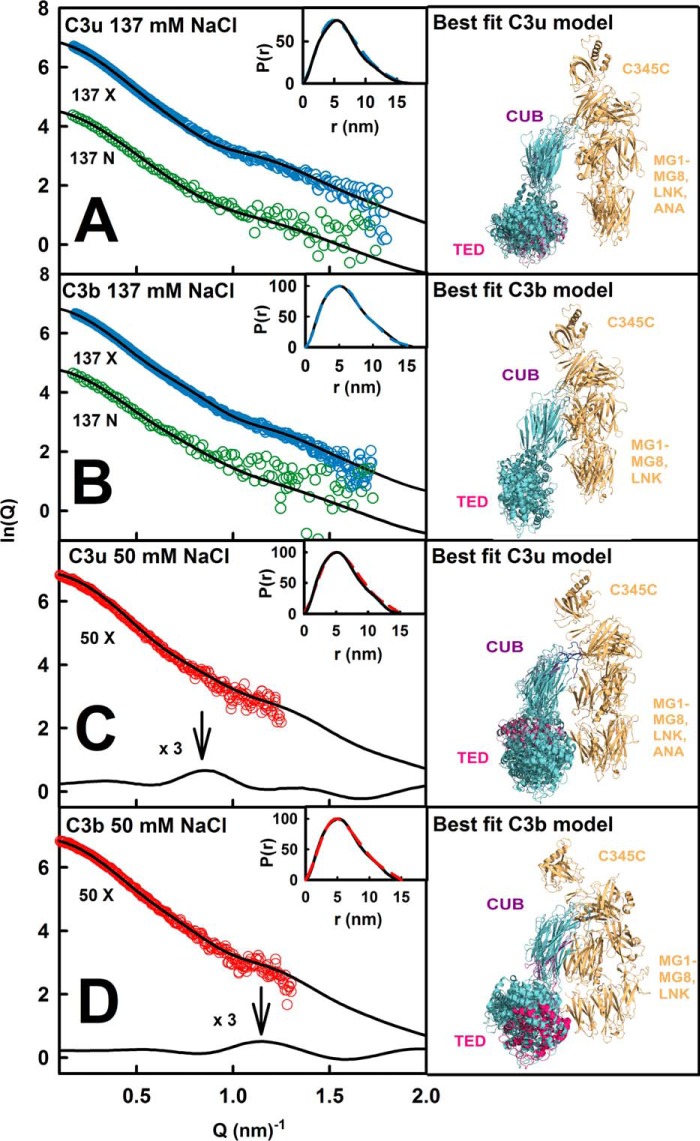
**Scattering modeling fits for C3u and C3b in three buffers.** *A*, the x-ray and neutron fits for C3u at 0.82–1.0 mg/ml in 137 mm NaCl buffer in light and heavy water are shown in *blue* and *green*, respectively. *B*, the x-ray and neutron fits for C3b at 0.52–1.0 mg/ml in 137 mm NaCl buffer in light and heavy water are shown in *blue* and *green*, respectively. *C*, the C3u x-ray curve in 50 mm NaCl buffer in light water was extrapolated to zero concentration. Subtraction of the fitted C3u x-ray curve in 137 mm NaCl buffer revealed a peak at 0.86 nm^−1^. *D*, the C3b x-ray curve in 50 mm NaCl buffer in light water was extrapolated to zero concentration. Subtraction of the fitted C3b x-ray curve in 137 mm NaCl buffer revealed a peak at 1.14 nm^−1^. The *insets* show the experimental (continuous) and modeled (*dashed*) x-ray *P*(*r*) curves. The *right-hand panels* show the 4–6 x-ray best fitted superimposed structures in the same orientation as Fig. 1, with the best fit TED domain shown in *crimson*.

Constrained modeling searches on C3u and C3b were performed to identify the position of TED-CUB relative to C3c that best fitted the four scattering curves, starting from a library of 4650 randomized structures (see “Experimental Procedures”). When the goodness of fit *R*-factors were compared with the 4650 modeled *R_g_* values (Fig. 9, *A–F*), a single minimum was observed in all six cases, showing that a single family of conformers best fitted the data. The lowest *R*-factors corresponded to models with *R_g_* values similar to the experiment. In all six fits, the best fit *R_g_* values were higher for C3u compared with C3b, indicating that C3u is more elongated than C3b. (i) The x-ray fits for C3u in 137 mm NaCl replicated earlier results (16). The addition of the new neutron fits showed that five of the nine best fit models were identical to the 10 x-ray best fit models. The *R*-factor was low at 3.3% (x-ray) and 4.4% (neutron) (Table 2, *B* and *C*). The calculated and experimental *I*(*Q*) and *P*(*r*) curves showed good visual agreement (Fig. 10*A*). The calculated *s*_20,*w*_^0^ value of 7.98 S agreed well with the experimental value of 8.03 S. The best fit models showed that the TED and MG1 domains were well separated by 6.0 ± 0.6 nm (x-ray) and 6.2 ± 0.6 nm (neutron) between their centers of mass. (ii) For C3u in 50 mm NaCl, the x-ray fits gave a low *R*-factor of 3.1% (Table 2*D*) with good visual agreement (Fig. 10*C*). The calculated *s*_20,*w*_^0^ value of 7.88 S agreed well with the experimental value of 8.10 S. Here the best fit models showed a TED-MG1 separation of 4.3 ± 0.4 nm, within error of that of 3.8 ± 0.4 nm in the C3b crystal structures. (iii) For C3b in 137 mm NaCl, five of the 9–10 best fit models from the x-ray and neutron fits were identical. Good visual curve fits were obtained (Fig. 10*B*). The best fit C3b model has *R*-factors of 2.2% (x-ray) and 5.7% (neutron) (Table 2, *B* and *C*), these being improved compared with those from the C3b crystal structures. The calculated *s*_20,*w*_^0^ value of 7.46 S agreed well with the experimental value of 7.40 S. The TED-MG1 separation was 5.2 ± 0.5 nm (x-ray) and 5.2 ± 0.5 nm (neutron), this being greater than in the C3b crystal structures. Our x-ray and neutron fits strongly indicate a single conformation for C3b, unlike earlier C3b modeling in 200 mm NaCl that suggested two different conformations (18). (iv) For C3b in 50 mm NaCl, the x-ray best fit C3b model had a low *R*-factor of 2.8% (Fig. 10*D* and Table 2*D*). The calculated *s*_20,*w*_^0^ value of 7.60 S agreed with the experimental value of 7.60 S. The TED-MG1 separation was 4.3 ± 0.4 nm, within error of the C3b crystal structures.

**FIGURE 11.**
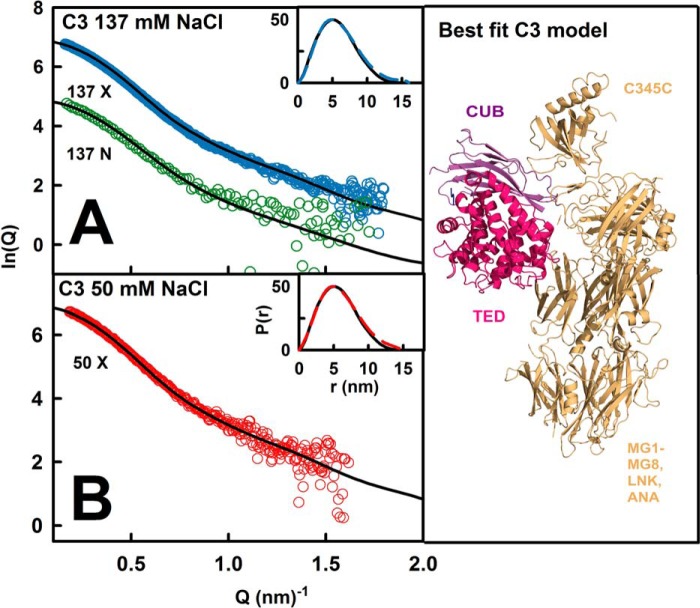
**Scattering modeling fits for C3 in three buffers.** *A*, the x-ray and neutron fits for C3 at 0.75–1.2 mg/ml in 137 mm NaCl buffer in light and heavy water are shown in *blue* and *green*, respectively. *B*, x-ray fit for C3 in 50 mm NaCl buffer in light water extrapolated to zero concentration. *C*, the best fit C3 structure is shown. For more details, see the legend to Fig. 8.

Control calculations showed that constrained modeling was able to replicate the C3 crystal structure. In fit searches, each of the TED and CUB domains was varied separately relative to the C3c region. Single *R*-factor minima were observed (Fig. 9, *G–I*). The best fit *R_g_* values were lower for C3 compared with C3u and C3b, indicating that C3 has the most compact structure. (i) The x-ray fits for C3 in 137 mm NaCl replicated earlier results (Fig. 11*A*) (16). Four of the eight neutron best fit models were the same as the x-ray best fit models. The *R*-factor was low at 3.1% (x-ray) and 5.1% (neutron) (Table 2, *B* and *C*). The calculated *s*_20,*w*_^0^ value of 8.57 S agreed with the experimental value of 8.49 S. (ii) X-ray fits for C3 in 50 mm NaCl showed that four of the eight best fit models were the same as in 137 mm NaCl, and the x-ray *R*-factor was 3.5% (Fig. 11*B* and Table 2*D*). The calculated *s*_20,*w*_^0^ value of 8.57 S agreed well with the experimental value of 8.50 S.

##### Mutagenesis of the TED-MG1 Binding Interface

**FIGURE 12.**
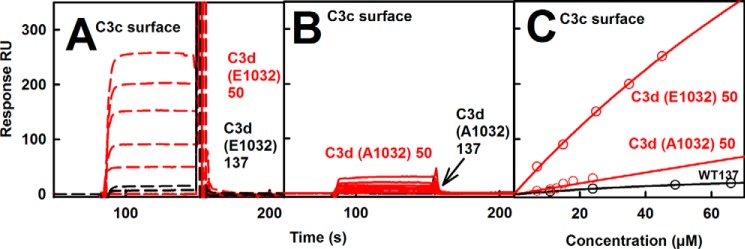
**Analysis of the Arg^102^–Glu^1032^ salt bridge using surface plasmon resonance.** *A* and *B*, wild type C3d (*E1032*) and mutant C3d (*A1032*) analytes were injected over amine-coupled immobilized C3c as ligand in 50 mm NaCl (*red*) and 137 mm NaCl (*black*) buffers. *C*, corresponding *K_D_* fits for 50 mm NaCl (*red*) and 137 mm NaCl (*black*). C3d (*E1032*) gave a *K_D_* value of 51 μm in 50 mm NaCl buffer.

In order to verify that the structural changes in C3b and C3u between 50 and 137 mm NaCl were attributable to the Arg^102^–Glu^1032^ salt bridge seen in the C3b crystal structure in 50 mm NaCl, this interaction was validated by surface plasmon resonance. When wild-type C3d was injected over immobilized C3c in 50 mm NaCl, a significant response was detected, leading to a *K_D_* value of 51 μm (Fig. 12*A*). In 137 mm NaCl, C3d binding to immobilized C3c was much weakened, with the *K_D_* value estimated to be in the millimolar range. This indicated the loss of this salt bridge in 137 mm NaCl. The reversed experiment with C3c injected over immobilized C3d showed the same outcome (not shown). To test whether the Arg^102^–Glu^1032^ salt bridge itself accounted for the observed separation of the TED-MG1 interaction in C3b and C3u in 137 mm NaCl, but not in 50 mm NaCl (above), the C3d E1032A mutant was used. Mutant C3d bound 10-fold less to C3c in 50 mm NaCl, and binding was not observed in 137 mm NaCl (Fig. 12*B*). The reversed experiment with the immobilized C3d mutant also showed no binding to C3c in either buffer.

## DISCUSSION

**FIGURE 13.**
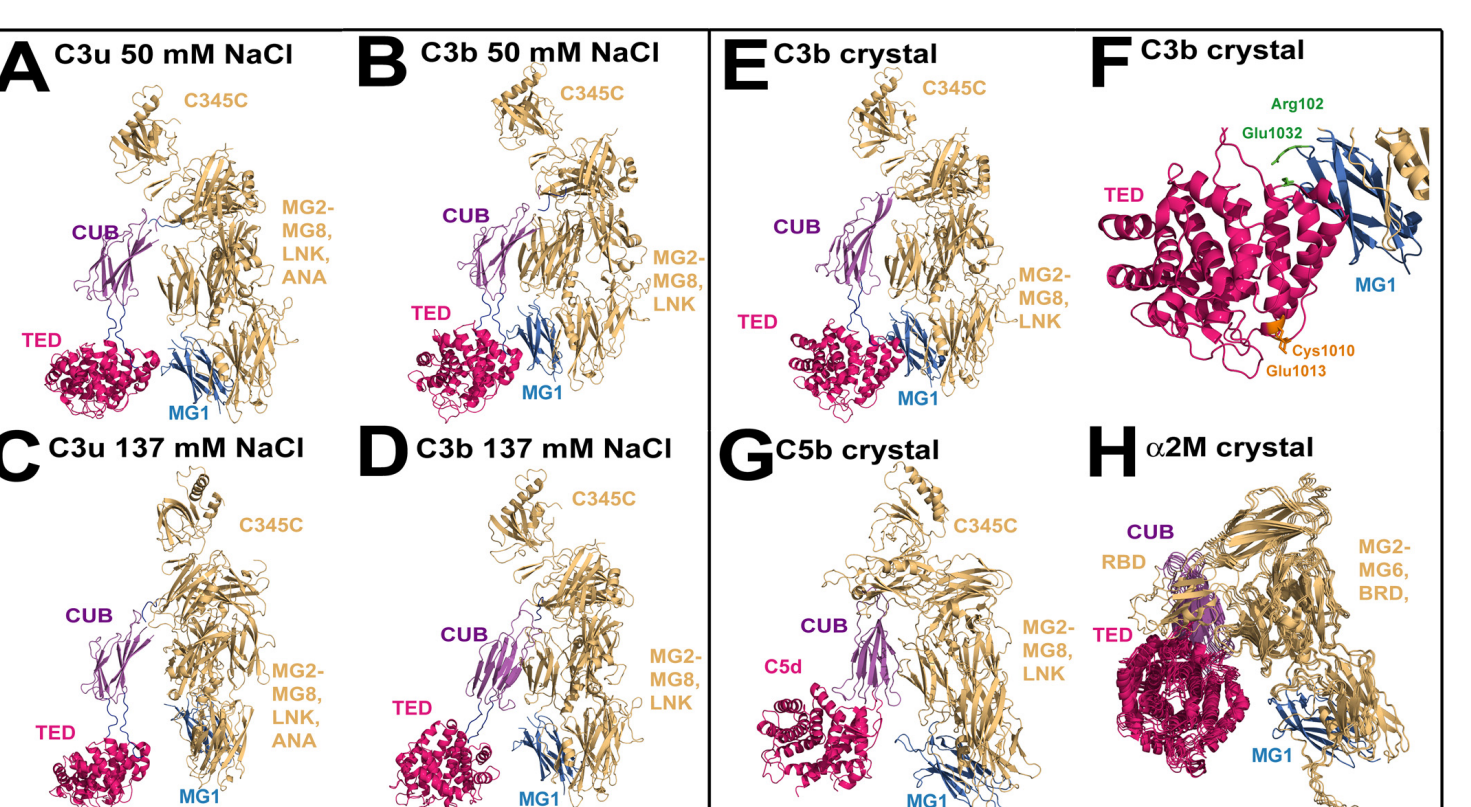
**Structures of C3b and C3u in 50 mm and 137 mm NaCl buffers.** *A* and *B*, best fit solution structures for C3u and C3b in 50 mm NaCl show that the TED (*crimson*) and MG1 (*blue*) domains are close to each other. *C* and *D*, best fit solution structures for C3u and C3b in 137 mm NaCl show that the TED (*crimson*) and MG1 (*blue*) domains have separated in this buffer. *E*, the C3b structure crystallized in 50 mm NaCl is similar to those in *A* and *B. F*, salt bridge interaction at Arg^102^ (MG1; *blue*) and Glu^1032^ (TED; *crimson*) is shown in *green*. The thioester (Cys^1010^ and Glu^1013^) is shown in *orange. G* and *H*, crystal structures for C5b in its complex with C6 (not shown) and the four superimposed monomers of active α_2_-macroglobulin. In these structures, the TED (*crimson*) and MG1 (*blue*) domains are also separated.

In physiological 137 mm NaCl buffer, our solution structures for C3b and C3u showed that the TED domain in C3b and C3u was separated by as much as 6 nm from the MG1 domain in the C3c region (Fig. 13, *C* and *D*). This result changes our understanding of the TED domain. It had been frequently assumed that the TED and MG1 domains of C3b remain connected to form a compact structure in solution (4); however, this structure is now seen to be an artifact of crystal packing in non-physiological conditions of 50 mm NaCl. Gratifyingly, our C3b and C3u solution structures in 50 mm NaCl (Fig. 13, *A* and *B*) agreed well with the C3b crystal structures, all four of which were crystallized in 50 mm NaCl buffer (Fig. 13*E*). This TED conformational change in 137 mm NaCl means that, mechanistically, C3b is now best viewed as a more reactive molecule than previously believed, with a mobile thioester group that readily binds to antigenic surfaces. If the TED-MG1 interaction was rigidly held together in C3b, this would restrict the ability of the thioester to bind to appropriate antigenic surfaces. These results may indicate a novel structural basis for the future design of improved complement inhibitors targeted against C3b in which the TED-MG1 domains become more strongly linked.

C3b forms a connected TED-MG1 structure in its regulatory complex with the Factor H SCR-1/4 (short complement regulator 1/4) domains (13). The stabilization of a less active and compact C3b conformation by Factor H is part of its regulatory mechanism, followed by the cleavage of C3b at its CUB domain by Factor I. The Arg^102^–Glu^1032^ salt bridge in C3b is required for this complex (Fig. 13*F*). Similar residues in the human C4 sequence occur at Arg^63^ and Glu^1032^ but not in the sequences of human C5 or α_2_-macroglobulin. Presumably, this salt bridge is conserved when a regulatory control mechanism became essential. In fact, separated TED-MG1 structures were seen to be physiologically important in the crystal structure of complement C5b (a C3b homologue) in complex with complement C6, which also showed a TED-MG1 separation of 5.4 nm (Fig. 13*G*) (20, 21). Separated TED-MG1 structures were also seen for methylamine-induced (active) α_2_-macroglobulin (19). α_2_-Macroglobulin is a tetramer of C3b-like structures that is formed through contacts between pairs of TED domains and pairs of MG3-MG4 domains. The TED-MG1 separation in α_2_-macroglobulin is 5.6–5.7 nm (Fig. 13*H*), similar to that of 5.2–6.2 nm in our C3b and C3u structures in 137 mm NaCl. An extended C3b structure was also observed in its complex with bacterial Efb-c, where the TED-MG1 separation was about 4.9 nm (18). For C3u, a further need for the Arg^102^–Glu^1032^ salt bridge may be related to the low intracellular ∼10 mm NaCl concentration within the hepatic cells that synthesize the complement proteins. Low salt concentrations would favor the functionally inactive structure if intracellular C3u is inadvertently formed, so that complement is not accidentally activated during its secretion.

Our finding that the TED-MG1 domains are separated in C3b in 137 mm NaCl explains the significance of the R102G polymorphism that distinguishes the major C3S and C3F allotypes. First, the loss of the Arg^102^–Glu^1032^ salt bridge in 137 mm NaCl means that the positively charged Arg^102^ side chain becomes fully solvent-exposed at the surface of C3S. This now explains why C3S migrates more slowly toward the anode than C3F during non-reducing SDS-PAGE; the crystal structure had predicted that Arg^102^ would be buried in solution. Second, our surface plasmon resonance studies showed that the Arg^102^–Glu^1032^ salt bridge is crucial for controlling the TED-MG1 separation (Fig. 12), and this is important for the regulatory C3b-Factor H SCR-1/4 interaction, which is stabilized by this salt bridge. The loss of this salt bridge in the C3F allotype is associated with age-related macular degeneration, leading to deposition of complement C3 in drusen, and with atypical hemolytic uraemic syndrome, leading to endothelial tissue damage in the kidneys. Excessive complement activation is involved in both pathogeneses, leading to greater inflammatory damage.

This study was made possible by advances in atomistic scattering modeling that successfully identified the compact and extended structures for C3u and C3b. Analytical ultracentrifugation clarified weak dimer formation in the scattering data that required correction. The advent of high throughput scattering data collection permitted detailed data analyses for all five C3 forms in three buffers, including sufficient data to permit the curves to be extrapolated to zero concentration. Complementary x-ray and neutron scattering curves were used for modeling (22). The quality of the curve fits were similar or improved compared with other complement proteins and antibodies (47). By scattering, the crucial TED structural movement in C3u and C3b between 50 and 137 mm NaCl was most clearly seen by a reproducible inflection following subtraction of the 137 and 50 mm NaCl curve fits (Fig. 10, *C* and *D*). This study is thus an advance on previous x-ray scattering of C3u and C3b (16, 18) that showed that C3u and C3b were more extended than the C3b crystal structures but did not explain the difference. The earlier use of 5% glycerol may have perturbed the C3b conformation through the promotion of excessive hydrogen bonding with glycerol (17, 49). As an alternative method, electron microscopy showed the existence of more extended or more compact structures for negatively stained C3b and C3u *in vacuo*; however, the context of these two structural forms was not identified (9).

Dimer formation was an unwanted feature of the C3 proteins but required consideration for accurate modeling analyses. Ultracentrifugation showed that C3d exhibited a monomer-dimer-tetramer equilibrium in 50 mm NaCl buffer but was monomeric in 137 mm NaCl (46). Two types of dimers occurred for C3 and C3u in 50 mm NaCl (16). The faster equilibrium was attributable to C3d-C3d (TED-TED) contacts, whereas the slower one was attributable to C3c-C3c contacts. The present study with five C3 proteins extended these observations. The TED domain in C3, C3u, and C3b was linked with pronounced concentration dependences only in 50 mm NaCl (Figs. 4 and 6). The resulting monomer-dimer dissociation constant *K_D_* values were 40–70 μm in both 50 and 137 mm NaCl for C3, C3b, C3u, and C3c. The *K_D_* value for C3d self-association was 25 μm in 50 mm NaCl and was not observable in 137 mm NaCl. These *K_D_* values showed that these monomer-dimer equilibria were not physiologically important at typical protein concentrations of about 5 μm. Nonetheless, dimer formation in the C3 proteins is important to consider in experimental studies if the buffer conditions or sample concentrations differ from their physiological ranges.

## Supplementary Material

Supplemental Data

## Footnotes

2 The abbreviations used are:

MG1–MG8: macroglobulin 1–8, respectively
CUB: C1r/C1s-UEGF-BMP1
TED: thioester-containing domain(s).
