# Supplementary material for: A Revised Mechanism for the Activation of Complement C3 to C3b: A MOLECULAR EXPLANATION OF A DISEASE-ASSOCIATED POLYMORPHISM
Source: J Biol Chem. 2014 Dec 8;290(4):2334–50. doi: 10.1074/jbc.M114.605691 (PMC4303685; doi:10.1074/jbc.M114.605691)
Supplement: Supplemental Data [file supp_290_4_2334__index.html]

A Revised Mechanism for the Activation of Complement C3 to C3b — Solution Structures of C3b and C3u — Supplemental Data 

# A Revised Mechanism for the Activation of Complement C3 to C3b

## Supplemental Data

**Files in this Data Supplement:**

- C3b 50 mM NaCl (.pdb, 903 KB) - C3b 50 mM NaCl
- C3b 137 mM NaCl (.pdb, 1.2 MB) - C3b 137 mM NaCl
- C3u 50 mM NaCl (.pdb, 1.2 MB) - C3u 50 mM NaCl
